# Non-invasive assessment of proarrhythmic risks associated with isoprenaline and the dietary supplement ingredient synephrine using human induced pluripotent stem cell-derived cardiomyocytes

**DOI:** 10.3389/fcvm.2024.1407138

**Published:** 2024-06-07

**Authors:** Xin Yuan, Tingting Yu, Zhang Zhang, Sen Li

**Affiliations:** School of Life Sciences, Beijing University of Chinese Medicine, Beijing, China

**Keywords:** *p*-synephrine (SYN), isoprenaline (ISO), adrenomimetic drugs, proarrhythmia, cardiovascular effect

## Abstract

**Background:**

There have been conflicting reports about the proarrhythmic risk of *p*-synephrine (SYN). To address this, human induced pluripotent stem cell-derived cardiomyocytes (hiPSC-CMs) combined with the microelectrode array (MEA) system have been utilized to assess arrhythmia risks, particularly in the context of adrenomimetic drugs.

**Aim:**

This study aims to determine whether MEA recordings from hiPSC-CMs could predict the proarrhythmic risk of adrenomimetic drugs and to investigate the cardiovascular effects and mechanisms of SYN.

**Materials and methods:**

We employed MEA recordings to assess the electrophysiological properties of hiPSC-CMs and conducted concentration-response analyses to evaluate the effects of SYN and Isoprenaline (ISO) on beating rate and contractility. A risk scoring system for proarrhythmic risks was established based on hiPSC-CMs in this study. ISO, a classic beta-adrenergic drug, was also evaluated. Furthermore, the study evaluated the risk of SYN and recorded the concentration-response of beating rate, contractility and the change in the presence or absence of selective β1, β2 and β3 adrenergic blockers.

**Results:**

Our results suggested that ISO carries a high risk of inducing arrhythmias, aligning with existing literature. SYN caused a 30% prolongation of the field potential duration (FPD) at a concentration of 206.326 μM, a change significantly different from baseline measurements and control treatments. The half maximal effective concentration (EC50) of SYN (3.31 μM) to affect hiPSC-CM beating rate is much higher than that of ISO (18.00 nM). The effect of SYN at an EC50 of 3.31 μM is about ten times more potent in hiPSC-CMs compared to neonatal rat cardiomyocytes (34.12 μM). SYN increased the contractility of cardiomyocytes by 29.97 ± 11.65%, compared to ISO's increase of 50.56 ± 24.15%. β1 receptor blockers almost eliminated the beating rate increase induced by both ISO and SYN, while neither β2 nor β3 blockers had a complete inhibitory effect.

**Conclusion:**

The MEA and hiPSC-CM system could effectively predict the risk of adrenomimetic drugs. The study concludes that the proarrhythmia risk of SYN at conventional doses is low. SYN is more sensitive in increasing beating rate and contractility in human cardiomyocytes compared to rats, primarily activating β1 receptor.

## 1 Introduction

*p*-synephrine (SYN), a natural alkaloid extracted from *Citrus aurantium* L. (commonly known as bitter orange or sour orange), is frequently used in dietary supplements for weight management and sports performance as an alternative to ephedra. SYN is also found in various citrus foods. However, its safety has been a subject of debate. The Food and Drug Administration (FDA) (1), Health Canada (2) and other institutions have reported numerous adverse events related to SYN, predominantly cardiovascular issues such as QT prolongation-induced exercise syncope, tachycardia, ventricular fibrillation, cardiac arrest, and sudden death (3). Several case reports and articles (4–10) suggest a link between SYN, bitter orange extract, and cardiovascular events, including an increased heart rate (11). This is further supported by animal studies (12, 13). In contrast, other investigations on SYN (14–17) report no observed cardiovascular effects. Moreover, while some studies (11, 18–20) indicate that SYN might accelerate heart rate, we hypothesize that this could be due to impurities (ephedrine/m-synephrine/N-methyltyramine) or its combination with caffeine. To determine the cardiovascular effects of SYN and its potential to cause arrhythmias, thorough safety assessments are essential.

Human induced pluripotent stem cell-derived cardiomyocytes (hiPSC-CMs) exhibit properties akin to adult cardiomyocytes (21, 22), such as the expression of crucial structural and functional genes, ultrastructural characteristics like Z disks and T-tubules, electrophysiological phenotypes, and ion channel functions (23), as well as mature excitation-contraction coupling (24, 25). Thus, hiPSC-CMs are increasingly used as renewable experimental materials and are anticipated to become a comprehensive tool for *in vitro* torsadogenic liability assessment (26). Despite the wide use of hiPSC-CMs in drug evaluation, a comprehensive methodology to accurately predict the arrhythmic risk posed by β-adrenergic agonists, including the widely used SYN, is notably lacking. This gap is critical, considering the widespread consumption of SYN-containing supplements and their potential cardiovascular risks. The multi-electrode array (MEA) system can record field potentials (FP) of spontaneously beating cardiomyocyte monolayers, analogous to clinical electrocardiogram (ECG). The field potential duration (FPD) is comparable to the QT interval, and can be used to analyze the repolarization times. Additionally, early afterdepolarization (EAD) and torsadogenic and arrest events are instrumental in assessing proarrhythmia risks (27–30). However, the methodology for predicting arrhythmia risks using hiPSC-CM and MEA is not yet fully established. While certain drugs like E4031 and Quinidine, that directly affect ion channels have been evaluated using this method, studies on β receptor agonist-related ligands are scarce. Unlike ion channel blockers that prolong the QT interval, isoprenaline (ISO) can shorten it. This shortening also poses arrhythmia risks. ISO, a full β-receptor agonist, has been shown in numerous studies to shorten the QT interval, thereby increasing arrhythmias risks. Despite its widespread use, the safety of SYN remains debated. Numerous studies, such as those by Stohs et al. (20) and Jordan et al. (2), have highlighted potential cardiovascular risks including increased heart rate and blood pressure, while others like Shara and Stohs (16) report no observed adverse cardiovascular effects under controlled conditions. Therefore, in this study, we assessed the arrhythmic risks of β receptor agonists.

In this study, we tested the classic beta-adrenergic receptor agonist ISO and the potential adrenomimetic drug SYN. To evaluate the arrhythmic risk of ISO and SYN, we measured FPD prolongation, EAD occurrence, and Torsades de pointes (TdP) incidences. We detected the concentration-response relationship of ISO and SYN in hiPSC-CMs and analyzed SYN's effects on two types of cells: hiPSC-CMs and neonatal rat cardiomyocyte (NRCM). We also assessed the inotropic effects of these drugs on hiPSC-CMs. To better understand the toxic mechanism of these drugs and explore their potential therapeutic value, we utilized inhibitors to identify their action targets. We hypothesize that through the application of MEA recordings and analysis of hiPSC-CMs, we can delineate the arrhythmic risk profile of SYN in a cell-based model, offering insights into its safety and mechanistic effects on the cardiovascular system. This study aims to bridge the existing knowledge gap by employing hiPSC-CMs to assess the proarrhythmic potential of SYN and compare its effects with ISO, thereby providing a clearer understanding of SYN's cardiovascular implications.

## 2 Method and materials

### 2.1 Experimental materials

HiPSC-CMs were obtained from HELP Therapeutics (Nanjing, China). DMEM/F-12 was obtained from Gibco (now part of Invitrogen, Carlsbad, CA). Fetal bovine serum (FBS) and horse serum (HS) were purchased from Corning-Cellgro (Manassas, Virginia, USA) and BIODEE (Beijing, China), respectively. SYN was purchased from MedChemExpress (New Jersey, USA). ISO and laminin were obtained from Sigma (Merck, USA). Collagenase Ⅱ and Penicillin-Streptomycin were sourced from Invitrogen (Carlsbad, CA). Trypsin was procured from Life Science (now part of Alphabet, USA). 5-bromo-2′-deoxyuridine (5-BrdU) was obtained from Solarbio (Beijing, China). To investigate the specific roles of β1, β2, and β3 adrenergic receptors in mediating the effects of ISO and SYN on beating rate and contractility, we employed selective blockers targeting each receptor subtype. The blockers were chosen based on their high selectivity for their respective target receptors, as demonstrated in previous studies (31, 32). By comparing the extent to which each selective blocker inhibits the changes in beating rate and contractility induced by ISO and SYN, we can infer the relative contributions of β1, β2, and β3 receptors to their mechanisms of action.

### 2.2 Cultivation and preparation of hiPSC-CMs

HiPSC-CMs were seeded on MEA chips and confocal dishes, which were pre-coated with 1% fibronectin. The cells were allowed to adhere for 48 h using recovery medium (Help Stem Cell Innovations). This was followed by replacing the medium with cardiac maintenance medium (Help Stem Cell Innovations). The medium was refreshed bi-daily. Approximately 7–10 days post-seeding, the spontaneous beating frequency of hiPSC-CMs stabilized at around 1 Hz. This maturation stage is critical as it closely resembles the functional characteristics of adult cardiomyocytes, making our model especially relevant for studying cardiac electrophysiology and drug responses.

### 2.3 Isolation and culture of NRCM

Neonatal Sprague Dawley rats, aged between 24 and 48 h, were euthanized rapidly by decapitation (33, 34). The thoracic cavity was opened under aseptic conditions to expose the heart, which was promptly removed and washed with pre-cooled D-Hanks solution. Subsequently, the hearts were separated from cardiopulmonary tissue and minced into approximately 1 mm^3^ pieces using scissors. These pieces were then transferred to a 50 ml centrifuge tube and digested with a 0.05% type II collagenase solution in a constant temperature water bath at 37°C with shaking at 220 rpm for 40 min. Post enzymatic digestion, the resultant single cell suspension was filtered through a cell strainer and centrifuged at 300 g for 5 min, followed by resuspension in DMEM/F12-based culture medium with 10% FBS, 5% HS, 1% penicillin/streptomycin, and 100 μM 5-BrdU. 5-BrdU was added to the culture medium as part of our protocol to inhibit fibroblast proliferation within the cardiomyocyte cultures. Differential centrifugation, based on the differing gravities of fibroblasts and myocytes (33), was used for cell separation. The isolated neonatal rat ventricular myocytes (NRVMs) were then cultured on 0.1% laminin-coated petri dishes. The experimental protocol received approval from the Ethics Committee of Beijing University of Chinese Medicine (BUCM).

### 2.4 Concentration-response design and EC50 calculation

The selection of concentration ranges for ISO and SYN was based on a comprehensive review of existing literature and preliminary dose-response studies. The chosen concentrations aimed to cover the therapeutically relevant range and beyond to assess both efficacy and safety. The process was as follows: Initially, the range of concentrations was broad and then progressively narrowed. In the first experiment, ISO was tested at concentrations of 0, 1 nM, 1 μM and 1 mM. It was observed to be effective within the range of 1 nM–1 μM, with stopping of beating at 1 μM. Consequently, for the second experiment, the concentrations were refined to 0, 0.001 nM, 0.01 nM, 0.1 nM, 1 nM, 10 nM, 100 nM and 1 μM based on the initial effective concentration range. Similarly, for SYN, the range was chosen to explore its potential arrhythmic effects at both low and high doses. The initial experiment tested concentrations of 0, 1 nM, 1 μM and 1 mM, finding effectiveness in the range of 1 μM–1 mM. The subsequent experiment, therefore, used a narrower concentration selection: 0, 0.3 μM, 1 μM, 3 μM, 27 μM, 81 μM, 243 μM and 729 μM. The half maximal effective concentrations (EC50) values were calculated using GraphPad Prism version 8.0.0 for Windows (GraphPad Software, San Diego, California USA).

### 2.5 Field potential (FP) recordings

The MEA system records the electrical activity of cardiomyocytes by measuring the field potentials across a grid of electrodes. We chose an MEA chip equipped with 120 electrodes because it provides extensive spatial resolution that allows for a detailed mapping of the electrophysiological properties across the cardiomyocyte monolayer. This choice was based on comparative analyses with other electrode configurations, which showed that 120 electrodes offer superior data fidelity essential for accurate measurement of subtle changes in cardiomyocyte activity. This technique mimics human cardiac electrophysiology by capturing the collective electrical signals from cardiomyocyte monolayers, thus providing insights into the arrhythmic potential of compounds tested. For this purpose, cardiomyocytes were cultured on a laminin-coated MEA chip for 7–10 days. The electrophysiological characteristics of the cardiomyocytes were recorded using the Multi-Channel Systems (MEA2100-120-System, Multi Channel Systems), under controlled conditions of 37°C and 5% CO_2_. The recorded signals were amplified and processed through a filter amplifier, then transferred to the Cardio2D+ software (Multi Channel Systems) for subsequent data acquisition and analysis.

### 2.6 FPD data analysis and estimation the risk of proarrhythmia

FPD is defined as the interval between the onset of the first sharp deflection and the second positive deflection peak in a waveform. For analysis, a stable recording signal is selected and observed for a duration of 30 s. Considering that β receptor activation can increase beating rate, Bazett's correction formula is employed to calculate the corrected field potential duration (FPDc). This correction involved adjusting FPD by the RR interval, using the formula: $\mathrm{FPDc}\;=\;\mathrm{FPD}/\sqrt{\mathrm{RR}}$. The absolute values of FPDc at each concentration are expressed as a percentage change from baseline values. To establish the equations for calculating the concentration that causes a 10% (FPDc10) and a 30% (FPDc30) change in FPDc, linear regression analysis is performed on the change in FPDc with increasing administration concentration.

The Relative torsadogenic risk of each drug is estimated based on scoring criteria outlined in Table 1. This relative risk is determined using FPDcF10, FPDcF30, and EAD values. The possible TdP risk scores ranged from 0 to 3 (35). A score of 0 is assigned when there is no change in FPD; a score of 1 when the percentage change in FPDc is between 10% and 30%; a score of 2 when the change exceeds 30%; and a score of 3 is assigned in the event of EAD, TdP, or cardiac arrest occurrences.

**Table 1:** A score system for the estimate of risk of a compound.

Score	0	1	2	3
FPDc change	No change	≥10%, <30%	≥30%	
EAD	−	−	−	+
TdP	−	−	−	+
Arrest	−	−	−	+

FPDc, corrected field potential duration; EAD, early afterdepolarization; TdP, torsades de pointes.

### 2.7 Contractility measurement

Cardiac contractility measurement was conducted using the brightfield (BF) channels of an inverted confocal microscope (FV3000, Olympus). The cardiomyocyte was placed in a specialized chamber maintained at 37 °C and 5% CO_2_. Sequential images of the cardiomyocyte contractions were captured using Olympus CellSens software. The analysis of these contractions was performed using the MUSCLEMOTION plugin in Image J (36).

### 2.8 Statistical analysis

Quantitative data in this study are presented as the mean ± standard error (SE). Statistical analyses were performed using GraphPad Prism version 9.0.0 for Windows (GraphPad Software, San Diego, California USA). Depending on the data characteristics, either one-way repeated measures ANOVA, one-way ANOVA, or paired t-tests were utilized, as appropriate. Statistical significance was established as *P *< 0.01 or *P* < 0.001.

## 3 Results

### 3.1 Torsadogenic risk assessment

**Figure 1:**
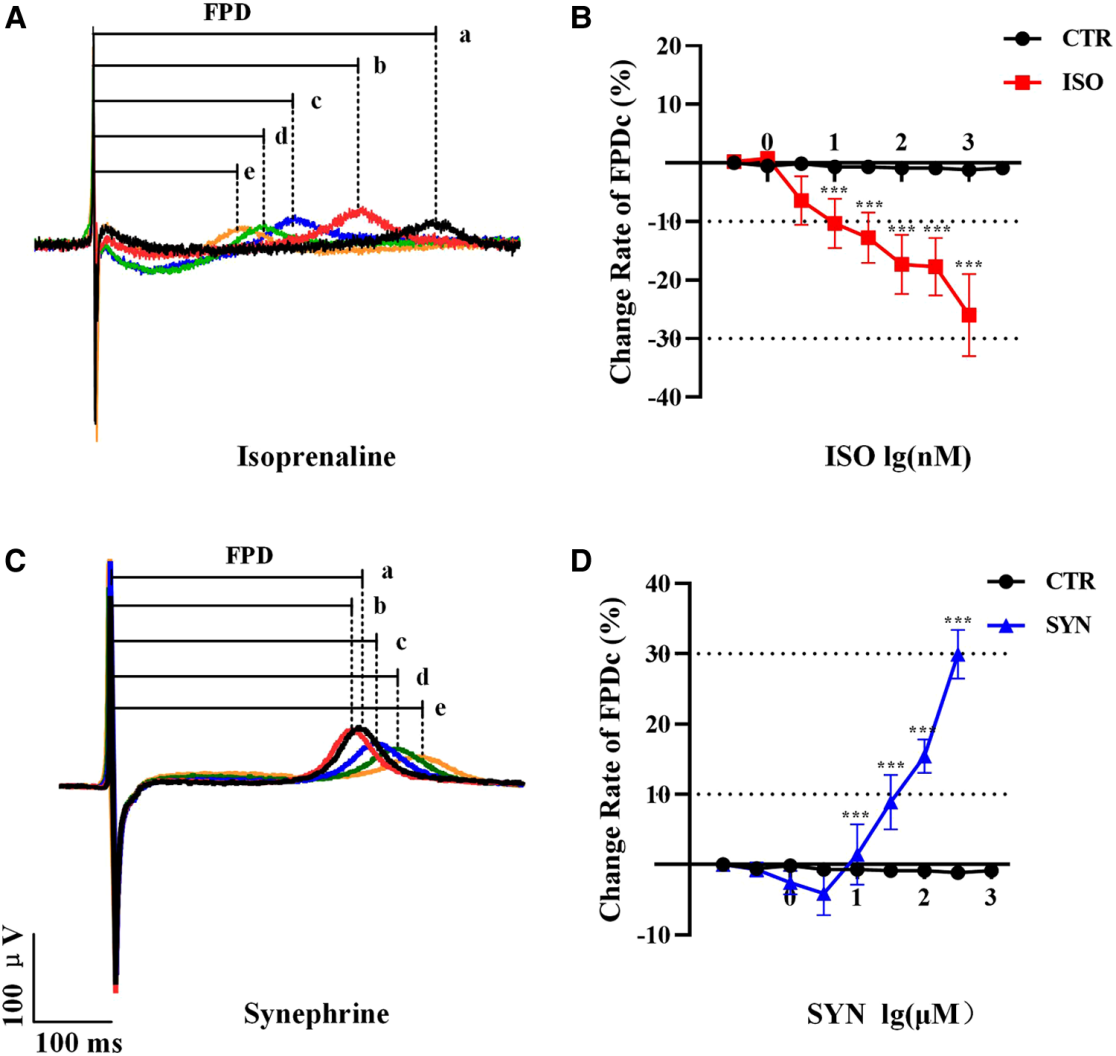
Representative MEA recordings, and the a, b, c, d, e represent increasing doses sequentially. The effect of ISO treatment on field potential duration (FPD) (**A**). FPDc change under ISO treatment (*n* = 10 electrode data per condition from four independent preparations, ***P < 0.001 vs control group) (**B**). The effect of synephrine treatment on FPD (**C**). Quantification the prolongation of FPDc with synephrine (*n* = 10 electrode data per condition from four independent preparations, ***P < 0.001 vs control group) (**D**).

**Figure 2:**
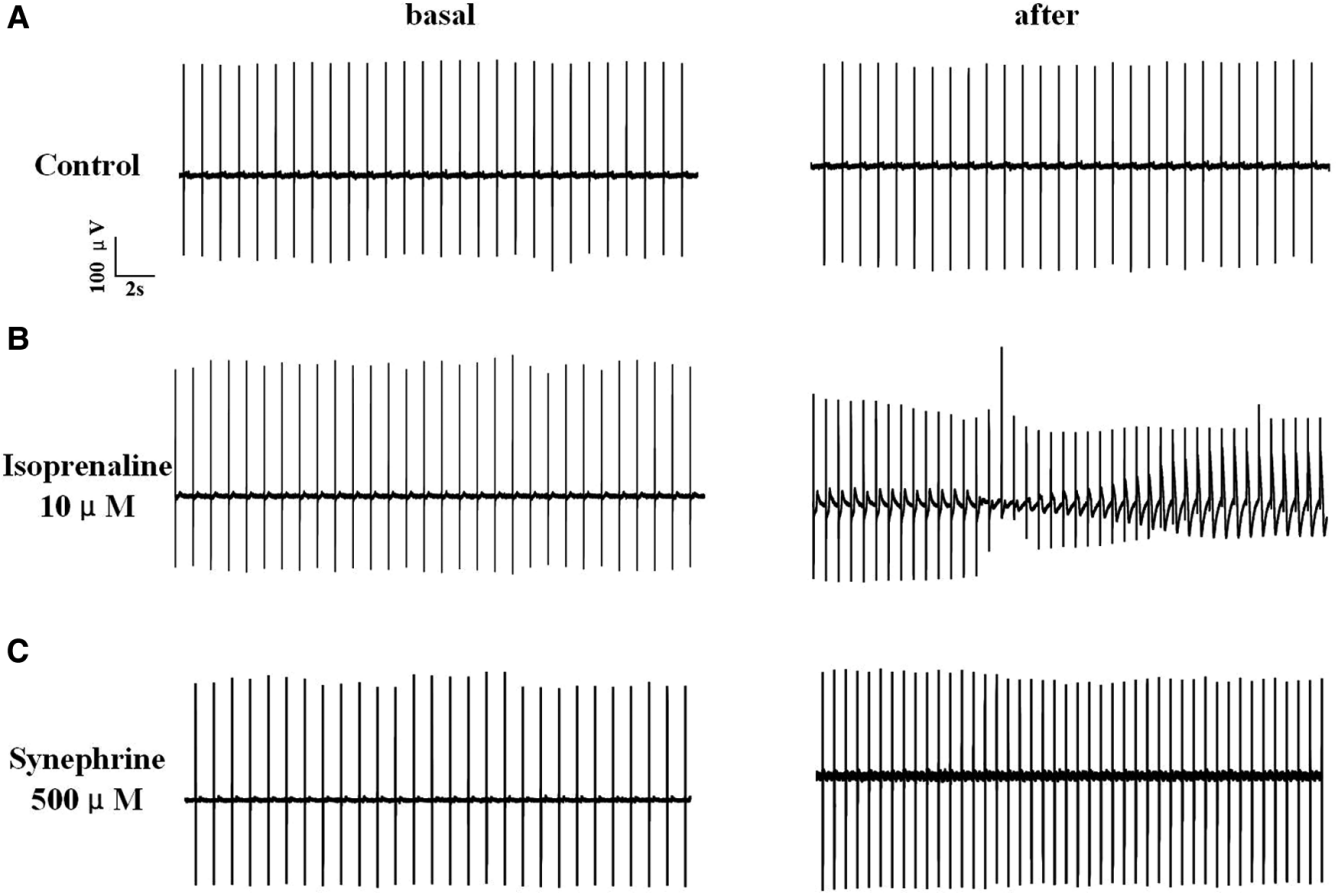
Electrical signals of hiPSC-CM recorded by MEA. The record of solvent control group (**A**). After treated with 10 μM isoprenaline, hiPSC-CM showed TdP-like arrhythmia (**B**). Under high concentration of 500 μM, synephrine increases heart rate without TdP-like curve (**C**).

The results indicated that FPDc varied with increasing concentrations of ISO and SYN. Notably, ISO shortened the FPDc within a certain concentration range (Figure 1A). The dose-dependent effects of ISO and SYN on FPDc, highlight their differential modulation of cardiomyocyte electrophysiology. When these changes induced by ISO were analyzed with Cardio2D+ software, and the subsequent linear fitting was performed using GraphPad software (Figure 1B). The concentrations of ISO causing a 10% (FPDc10) and 30% (FPDc30) reduction in FPDc were calculated, with the findings presented in Table 2. Conversely, SYN treatment generally led to the elongation of FPDc, demonstrated in Figure 1C. The progressive increase in FPDc with the increasing concentrations of SYN is shown in Figure 1D. The calculated concentrations of SYN that induce a 10% and 30% elongation of FPDc were 206.326 μM and 681.48 μM, respectively, detailed in Table 2. However, at an accumulated ISO concentration of 1 μM, TdP-like changes in cardiomyocyte electrical signals were noted in Table 2 and Figure 2. The significant shortening of FPDc by ISO underscores its proarrhythmic potential, while the more modest changes induced by SYN suggest a lower risk at clinical doses.

**Table 2:** Effects of ISO and SYN on FPDc, occurred event, and TdP in hiPSC-CM.

Compound	ETPC (μM)	Conc. FPDc10 (μM)	Conc. FPDc30 (μM)	Conc. occurred event			Highest Conc. with no event (μM)	TdP risk score
				EAD (μM)	TdP (μM)	Arrest (μM)		
Isoprenaline	0.002	0.036	3.988	N.D.	1	10	0.1	3
Synephrine	0.060	206.326	681.480	N.D.	N.D.	N.D.	1,000	0

ETPC, effective therapeutic plasma concentration; Conc, drug concentration; FPDc10, concentration inducing change of field potential duration by 10%; FPDc30, concentration inducing change of field potential duration by 30%; N.D., not detected.

### 3.2 Concentration-response of beating rate to ISO and SYN

**Figure 3:**
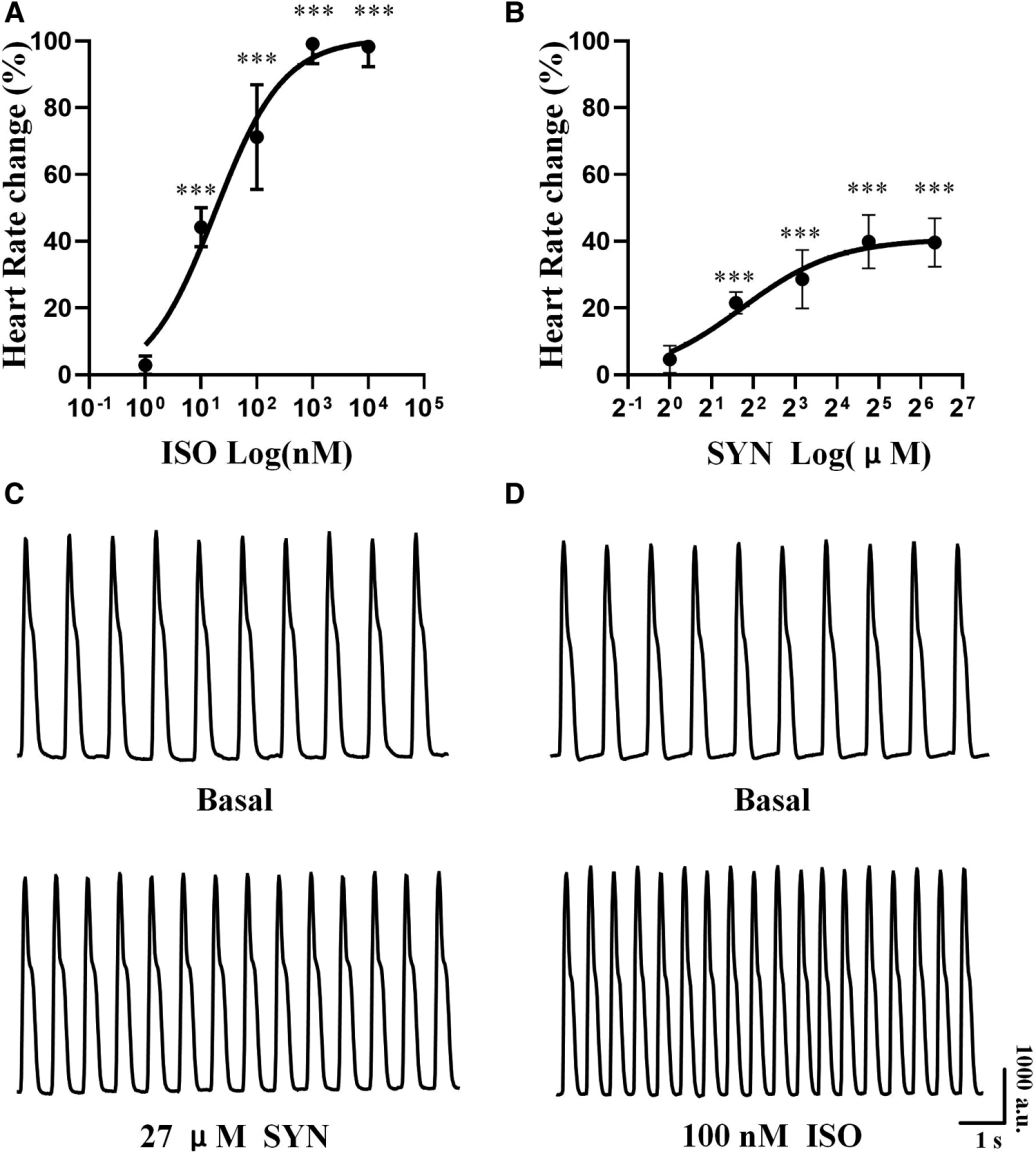
Cumulative concentration-response curve to isoprenaline (ISO) and synephrine (SYN) in hiPSC-CM. The percentage change in heart rate at each concentration (**A**, **B**). Representative curve of hiPSC-CM spontaneous contraction before and after exposure to 100 nM ISO and 27 μM SYN (**C**, **D**).

Both ISO and SYN were administered cumulatively to hiPSC-CMs, resulting in an increase in the beating rate of cardiomyocytes. The concentration of ISO was varied in a ten-fold gradient ranging from 1 to 10^4^ nM, whereas the SYN concentration was adjusted in three-fold increments from 1 to 81 μM. The increase in cardiomyocytes' beating rate was proportional to the cumulative concentration of ISO and SYN (Figure 3). The effect of ISO is characterized by an EC50 value calculated at 18.00 nM (95% CI: 8.825–40.30 nM) (Table 3).

**Table 3:** Compare two drug response in hiPSC-CM.

Drug	EC50	95% CI	HRM
Isoprenaline	0.018 μM	0.008825–0.0403 μM	100.60 ± 5.95%
Synephrine	3.314 μM	1.751–9.806 μM	40.74 ± 7.27%

hiPSC-CM, human induced pluripotent stem cell derived cardiomyocyte; NRCM, neonatal rat cardiomyocyte; 95% CI, 95% confidence interval; HRM, change of heart rate at maximal.

Conversely, the concentration-response curve for SYN on hiPSC-CMs yielded an EC50 value of 3.31 μM (95% CI: 1.751–9.806 μM). These effects are further compared to NRCMs in Table 4, where the EC50 value for NRCMs was found to be 34.12 μM (95% CI: 24.66–49.37 μM). The graphical representation of SYN's effects on cardiomyocytes is illustrated in Figure 3.

**Table 4:** Compare drug response in two cell types.

Cell type	Synephrine		HRM
	EC50 (μM)	95% CI	
hiPSC-CM	3.314	1.751–9.806	40.74 ± 7.27%
NRCM	34.12	24.66–49.37	40.43 ± 8.78%

hiPSC-CM, human induced pluripotent stem cell derived cardiomyocyte; NRCM, neonatal rat cardiomyocyte; EC50, the half maximal effective concentration; 95% CI, 95% confidence interval; HRM, change of heart rate at maximal.

### 3.3 Positive inotropic response to ISO and SYN

**Figure 4:**
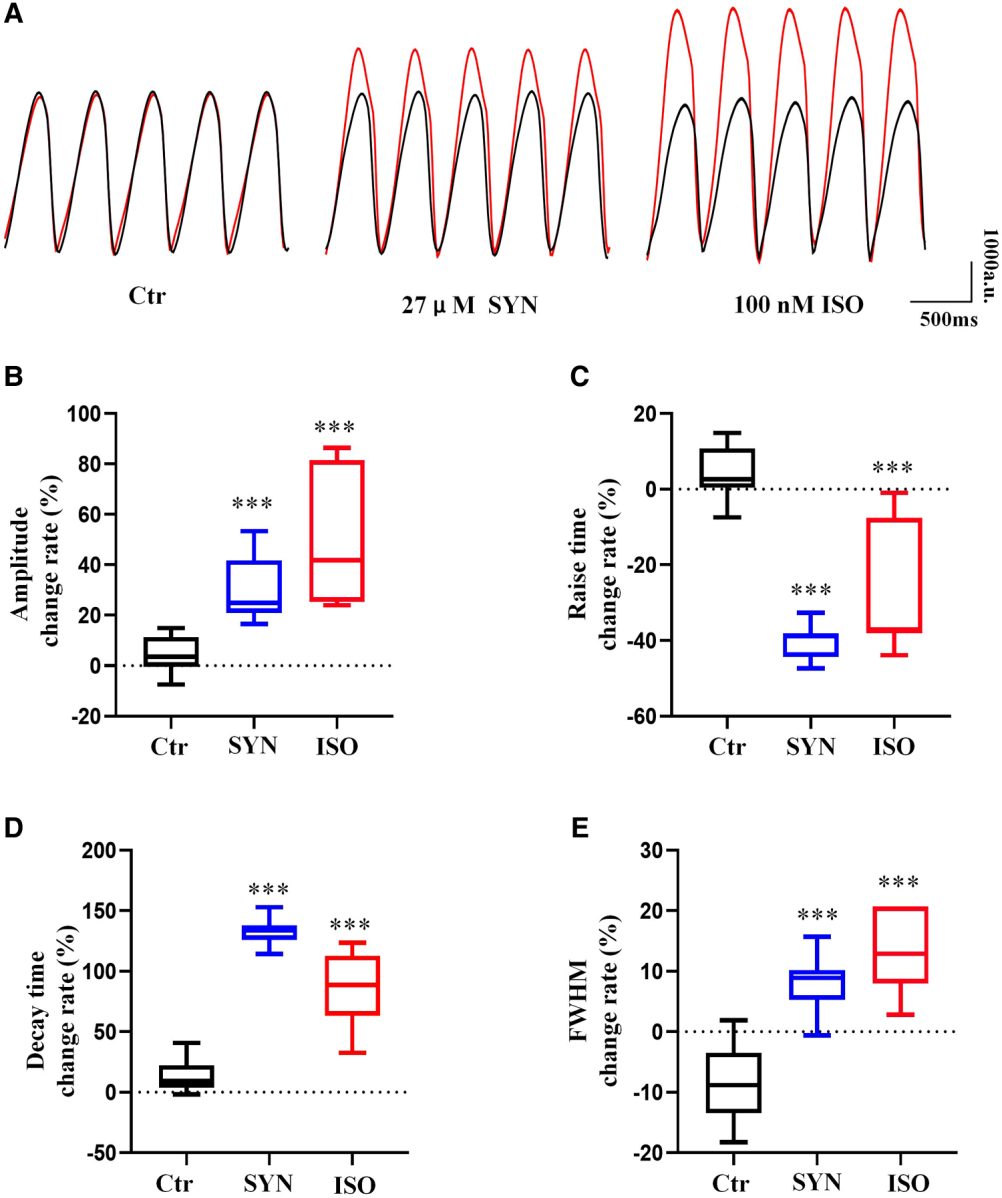
Cardiomyocytes recorded under 2 Hz field stimulation. The parameters of the beat are recorded by microscopic bright field mode and analyzed using MUSCLEMOTION software (**A**). The result of the presentation was the percentage change relative to the baseline. (**B**) Peak amplitude. The cell contraction is depicted by the displacement of the pixels, and the units are expressed in “a.u”. (**C**) Peak rise time. (**D**) Peak decay time. (**E**) Full width at half maximum. The symbol “***” represents a significant difference compared to control (*P* < 0.001).

The effect of ISO and SYN on cardiomyocyte contractility was shown in Figure 4A. To determine the effect of ISO and SYN on contraction functionality, four key parameters were analyzed: contraction amplitude, rise time (time from baseline to peak), decay time (time from peak to baseline), and full width at half maximum (FWHM). At a concentration of 100 nM, ISO significantly (*P* < 0.001) increased the contraction amplitude. SYN at 27 μM also resulted in an increase of contraction amplitude (Figure 4B). Both compounds reduced the rise time (Figure 4C). Conversely, both ISO and SYN significantly increased the decay time (Figure 4D). Furthermore, the FWHM for ISO and SYN also increased (Figure 4E).

### 3.4 Response dependence of the β-adrenoceptor subtype

**Figure 5:**
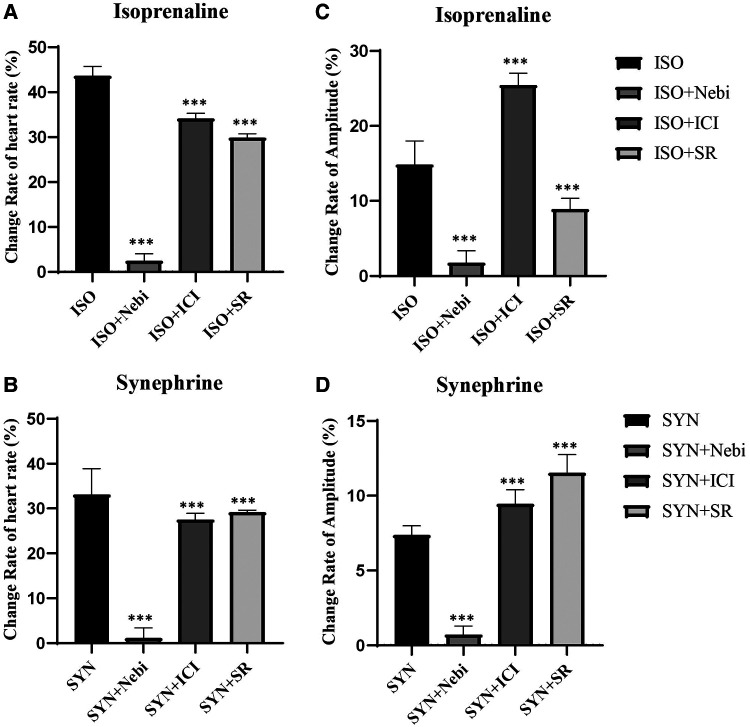
Changes in heart rate and contractility in the presence of the 10 nM β1 receptor selective blocker nebivolol, the 10 nM β2 selective receptor blocker ICI118551, and the 50 nM β3 selective receptor blocker SR59230. The Y axis is the rate of change compared to the basal heart rate (**A**, **B**) and contractility (**C**, **D**). SYN, synephrine; ISO, isoprenaline; Nebi, nebivolol; ICI, ICI118551; SR, SR59230.

Research into the specific roles of β1, β2, and β3 adrenergic receptors in modulating beating rate and contractility was performed through studies employing selective blockers. The results revealed that blockers targeting either β2 or β3 adrenergic receptors were unable to completely negate the increase in beating rate and contractility induced by ISO. In contrast, the application of β1 receptor blockers almost entirely abolished these effects, aligning with findings from previous reports. The effect of SYN mirrored some aspects of ISO's effects. When β1 receptor blockers were used, the influence of SYN on beating rate and contractility was almost entirely neutralized. This indicates that SYN likely to accelerate beating rate predominantly by stimulating β1 receptors (Figures 5). This observation implies that β2 and β3 receptors might play a secondary role in the modulation of SYN's effects.

## 4 Discussion

**Figure 6:**
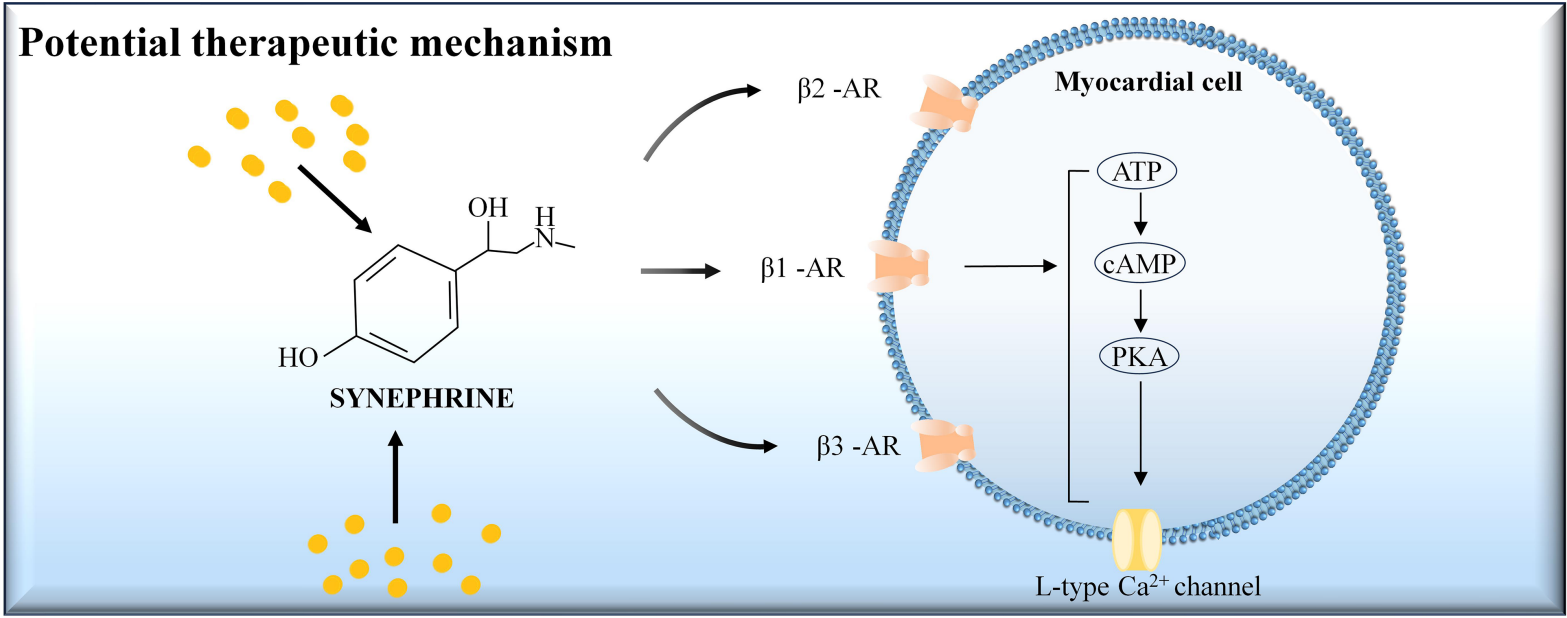
The potential therapeutic mechanism of synephrine on myocardial cells. Synephrine acts on beta-adrenergic receptors (β1-AR, β2-AR, and β3-AR) located on the myocardial cell membrane. Activation of these receptors stimulates the conversion of ATP to cAMP, which in turn activates protein kinase A (PKA). PKA phosphorylates the L-type Ca^2+^ channels, leading to increased calcium influx into the cell. This cascade enhances myocardial contractility and heart rate.

This study assesses the impact of ISO and SYN on arrhythmia risk and their underlying mechanisms, highlighting the potential of hiPSC-CM and MEA in predicting the risk of TdP induced by adrenomimetic drugs. SYN has a lower risk of arrhythmia at standard doses compared to ISO. Our findings also indicated that SYN's cardiovascular effects, including increased beating rate and myocardial contractility by directly stimulating β1, β2, and β3 receptors, are akin to those of ISO. The potential therapeutic mechanism of SYN on myocardial cells was presented in Figure 6. However, SYN's maximum influence on myocardial contractility and beating rate is less pronounced than ISO's. Notably, SYN demonstrates higher sensitivity in hiPSC-CMs compared to rat cardiomyocytes. The study suggests that ISO, a classic adrenomimetic drug, carries a higher risk of arrhythmia, whereas SYN has a lower risk of arrhythmia at clinical doses. Arrhythmia risk with SYN escalates at doses above 200 μM, significantly higher than the clinical dose. ISO has been clinically reported to have a higher risk of arrhythmia due to the shortening of the QT interval and the induction of TdP (37), which is consistent with our findings. Our study has investigated the SYN's arrhythmia risk in human cardiomyocytes, offering valuable insights for future research. In adrenomimetic drug studies, both shortening and prolonging of the FPD are noted (38), correlating with increased clinical arrhythmia risks. Thus, attention should be given not only to the prolongation of FPD, but also to arrhythmia risks from drug-induced FPD shortening. The International Council for Harmonisation (ICH) guidelines address arrhythmia risks associated with long QT intervals and drug-induced QT interval prolongation. The Comprehensive *In vitro* Proarrhythmia Assay (CIPA) project evaluates the risk of arrhythmia based on FPD prolongation, significantly benefiting preclinical cardiac safety assessment (39). However, current literature often overlooks the shortening of repolarization time by drugs, potentially leading to false-negative assessments of arrhythmia risks in drugs that shorten FPD. This study emphasizes the importance of monitoring FPD changes, and we have refined the comprehensive scoring system, contributing to the literature and reducing false-negative results to some extent. Nevertheless, further research with drugs known to have positive effects is necessary to improve the accuracy of the assessment.

The study further indicates that SYN is more reactive to human-derived cardiomyocytes than to rat-derived cardiomyocytes. Remarkably, SYN's EC50 on human-derived cardiomyocytes is approximately tenfold different from that on neonatal rat cardiomyocytes. This finding contrasts with the previous report of phenylephrine's heightened sensitivity in rodents over humans (40). This discrepancy underscores that clinical reports may not always align with rodent study outcomes, especially regarding SYN's cardiovascular effects. Therefore, caution is advised when extrapolating rat study findings to human arrhythmia risk assessments. This underscores the value of hiPSC-CMs as preclinical experimental models. The observed changes in rise time and decay time induced by SYN and ISO may be attributed to their effects on calcium handling in cardiomyocytes. The reduced rise time suggests an increased rate of calcium release from the sarcoplasmic reticulum, leading to faster contraction. Conversely, the prolonged decay time indicates a slower rate of calcium reuptake into the sarcoplasmic reticulum, resulting in a prolonged relaxation phase. These effects are likely mediated through the activation of β-adrenergic receptors, which are known to modulate calcium cycling in cardiomyocytes via the cAMP/PKA signaling pathway (41). Further studies investigating the specific effects of SYN and ISO on calcium-handling proteins, such as ryanodine receptors and SERCA, could provide a more detailed understanding of the mechanisms underlying the observed changes in contractility parameters.

Interestingly, our findings contradict previous reports that claim SYN scarcely binds to β1 receptors and lacks cardiovascular effects (42). This discrepancy may be attributed to variations in research materials and dosages used. Our data reveal that SYN's EC50 is much higher than that of ISO. Therefore, we hypothesize that SYN may act as a partial agonist. This potential partial agonism could be influenced by SYN's chemical structure, notably the absence of alkyl substitution on the α carbon in its phenylethanolamine side chain and the presence of a single p-hydroxyl group in its benzene ring. These structural characteristics likely affect SYN's receptor binding and pharmacokinetics, resulting in its comparatively lower efficacy relative to ISO. Contrary to some reports suggesting SYN does not significantly activate β1 and β2 receptors or has low affinity for them (31, 43). Our study indicates SYN may stimulate cardiomyocyte beating rate and contractility by activating β1, β2, and β3 receptors. Previous research often utilized animal cells or organs, such as guinea pig atrium and trachea (44). SYN has been noted to activate β3 receptors, promoting fat breakdown. Given the increased expression of β3 receptors in heart failure cardiomyocytes (45), they could be a viable target for treatment. The differential responses elicited by ISO and SYN could be partly attributed to their distinct structural configurations and their consequent interactions with β-adrenergic receptors. ISO, with its classic phenethylamine structure, is known to have a high affinity for both β1 and β2 receptors, which can result in pronounced cardiostimulatory effects. On the other hand, SYN possesses a similar but not identical structure, with subtle differences that may influence receptor binding and efficacy. The partial agonism of SYN could explain the more tempered physiological response when compared to the full agonist activity of ISO. Further research into the binding kinetics and second messenger systems activated by these compounds could elucidate the nuances of their interactions with cardiac cells and the resulting electrophysiological effects. SYN's capability to increase heart rate suggests its potential in treating bradycardia. While ISO is known for its robust agonistic effects on β-adrenergic receptors, SYN, with its lower arrhythmogenic potential and moderate effects on heart rate and contractility, may offer therapeutic benefits for chronic bradycardia.

The distinct pharmacological profile of SYN could have implications for its use in clinical settings where moderate increases in contractility are desired, highlighting their ability to enhance contraction amplitude. Notably, SYN is proposed to have therapeutic potential in the treatment of chronic heart failure, especially in combination with slow arrhythmia. Generally, β-blockers are often recommended in the treatment guidelines for heart failure, as β-receptor activation accelerates heart rate, increasing myocardial oxygen consumption and potentially exacerbating heart failure. In advanced heart failure patients with bradycardia, SYN, as a partial agonist, could offer certain advantages. Partial agonists exhibit minimal physiological effects post-receptor binding. They also function antagonistically in the presence of full agonists or at high concentrations. SYN's treatment can accelerate heart rate and increase myocardial contractility without excessively increasing heart rate. Moreover, SYN's partial agonist nature may counteract physiological hormones like epinephrine or β-receptor agonists, avoiding excessive cardiac load for a more balanced therapeutic effect. Consequently, SYN shows promise for heart failure treatment, though *in vivo* studies are imperative for further validation.

It is worth noting that the concentrations of SYN and ISO used in our *in vitro* study are higher than their clinically relevant doses. The discrepancy between our experimental concentrations and clinical doses can be attributed to several factors. First, *in vitro* studies directly expose cardiomyocytes to the compounds, whereas *in vivo* administration requires consideration of pharmacokinetic processes such as absorption, distribution, metabolism, and excretion (46). Second, our study aimed to investigate the proarrhythmic potential of SYN and ISO, necessitating the use of a wide concentration range to fully assess their safety profiles. Clinical therapeutic doses are typically lower than those causing severe adverse effects. Third, hiPSC-CMs used in our study might have different drug sensitivities compared to human adult cardiomyocytes *in vivo* (47). Lastly, the primary goal of our study was to compare the relative potencies of SYN and ISO and elucidate their proarrhythmic mechanisms, rather than determining clinically equivalent doses. Further studies are needed to extrapolate our findings to clinical settings and establish safe and effective dosing regimens for SYN and ISO. The observed effects of SYN and ISO on hiPSC-CMs, particularly the changes in beating rate, contractility, and proarrhythmic potential, are likely to be clinically significant. These *in vitro* findings provide valuable insights into the potential cardiovascular risks associated with the use of these compounds, especially at high doses or in susceptible individuals.

While the use of hiPSC-CMs presents a robust *in vitro* model for studying cardiomyocyte behavior under drug exposure, the extrapolation of these findings to *in vivo* human heart physiology warrants a cautious approach. HiPSC-CMs, while closely resembling human cardiomyocytes, are not perfect replicas. Therefore, additional research is needed to understand the reasons behind the varied FPD responses to adrenomimetic drugs like SYN and ISO, the differential dose responses of SYN in humans and rats, and the unexplored *in vivo* cardiovascular effects of SYN, as well as its therapeutic potential for bradycardia and heart failure. Although hiPSC-CMs express vital cardiac markers and replicate key aspects of adult cardiomyocyte function, hiPSC-CMs may not entirely capture the complex interplay of cellular interactions, mechanical forces, and systemic factors present in the whole heart. The microenvironment in a living organism, including neurohormonal modulation, shear stress from blood flow, and the multicellular architecture of the heart, can significantly influence drug response. Consequently, while the proarrhythmic risks identified in this study provide valuable insights, they represent a facet of the overall cardiotoxicity profile that must be validated through comprehensive *in vivo* studies. Further investigations utilizing animal models and clinical trials are imperative to confirm the translatability of the proarrhythmic potential of SYN and ISO observed in hiPSC-CMs.

## 5 Conclusion

Our findings indicate that ISO poses a higher risk of arrhythmia, whereas SYN demonstrates a lower risk of arrhythmia at clinical doses. In addition, SYN is shown to increase beating rate and myocardial contractility by directly activating β1, β2 and β3 receptors. This suggests SYN may have significant clinical utility in the treatment of conditions like bradycardia and complex heart failure, highlighting the need for further research in these areas.

## Data Availability

The original contributions presented in the study are included in the article, further inquiries can be directed to the corresponding author.
